# Effects of Drought Stress on Peramine and Lolitrem B in *Epichloë*-Endophyte-Infected Perennial Ryegrass

**DOI:** 10.3390/life12081207

**Published:** 2022-08-08

**Authors:** Weihu Lin, Chengfen Gao, Jianjun Wang, Wenbo Xu, Meining Wang, Miaomiao Li, Bihua Ma, Pei Tian

**Affiliations:** 1 State Key Laboratory of Grassland Agro-Ecosystems, Key Laboratory of Grassland Livestock Industry Innovation, Ministry of Agriculture and Rural Affairs, College of Pastoral Agriculture Science and Technology, Lanzhou University, Lanzhou 730020, China; 2 Institute of Rural Development, Gansu Provincial Academy of Social Sciences, Lanzhou 730071, China; 3 Gansu Grassland Technical Extension Station, Lanzhou 730010, China

**Keywords:** *Lolium perenne*, endophytes, alkaloids, drought stress

## Abstract

Perennial ryegrass (*Lolium perenne*) infected by *Epichloë* endophytes contains alkaloids that are responsible for toxicosis in many countries. Drought may greatly affect the alkaloids contents of symbionts. The E+ perennial ryegrass was grown in pots with different soil moisture conditions (15%, 30%, 45% and 60% relative saturation moisture content, RSMC) for four months in a greenhouse of Lanzhou University, and then, the aboveground tissues were collected. The levels of peramine and lolitrem B in all plant samples were determined. The results showed that the drought stress significantly (*p* < 0.05) increased the peramine concentrations of perennial ryegrass but did not affect the lolitrem B concentrations. In addition, the drought stress significantly (*p* < 0.05) reduced the plant height and dry matter of perennial ryegrass. In conclusion, drought stress affects the peramine concentration in the perennial ryegrass–endophyte symbiont but may not affect the lolitrem B concentration.

## 1. Introduction

Perennial ryegrass (*Lolium perenne*) is one of the widely cultivated forage and turf grass species due to its desirable agronomic performance in temperate climates [1]. Perennial ryegrass often forms mutualistic associations with *Epichloë* endophytes [2]. Universally, the asexual (e.g., *Epichloë festucae* var. *lolii*) *Epichloë* endophytes can infect perennial ryegrass [3]. Endophytes can increase the biotic and abiotic stress tolerance of the host grasses [4,5]. Possibly, this improvement comes from the perennial ryegrass–endophytic fungal symbiont’s production of lolitrem B and peramine alkaloids [6].

Over the last 30 years, the categories, mechanisms and synthesis of alkaloids have been examined and investigated. Four alkaloid categories have been identified: indole diterpenes, ergot alkaloids, pyrrolopyrazine and pyrrolizidine [7]. Indole diterpenes reduce the growth and development of some invertebrates [8]. Ergot alkaloids have also been identified as being a deterrent and/or toxic to an array of insect groups [9]. Pyrrolopyrazine alkaloids are a known insect feeding deterrent, which has no known activity against mammalian herbivores [10]. Pyrrolizidine alkaloids are also potent anti-invertebrate metabolites, and they can act as overt metabolic toxins (antibiosis) or feeding deterrents (antixenosis) [10]. Among them, lolitrem B, ergovaline, peramine and loline play important functions in the grassland ecosystem.

Several factors affect alkaloid content produced by endophytes, including the endophyte genotype [11], plant genotype [12,13], seasonal changes [14] and environmental conditions including soil moisture and temperature et al. [15,16]. Findings by Hahn demonstrated that the *Epichloë* endophyte can confer protection to perennial ryegrass from water stress and that the levels of alkaloids changed in response to this abiotic stress [17]. Repussard recorded that alkaloid concentrations were strongly influenced by plant distribution and climatic factors [18]. Detecting the presence and concentration of alkaloids in grasses infected with *Epichloë* spp. can provide an estimate of possible toxicity risks for livestock [19]. Therefore, it is very important to detect the concentration of alkaloids in symbionts under the different soil moisture conditions.

In the present study, our objective was to study the effects of varying soil moisture levels on the concentration of lolitrem B and peramine in *Epichloë*-endophyte-infected perennial ryegrass grown in pots.

## 2. Materials and Methods

### 2.1. Plant Material

Seeds of perennial ryegrass were obtained from the Bai Green International Grass Industry Co. Ltd. (Chaoyang, Beijing, China) [www.evergreen-international.com (accessed on 5 February 2015)]. These seeds were harvested from the same parent plant, and there were no genotype differences. In June 2015, healthy seeds were selected and planted into four seedling trays. Each tray contained 60 wells, which were filled with sterilized black soil [https://mjlcaotantu.cdgtw.net (accessed on 5 February 2015)]. When seedlings grew into 3–4 tillers, endophyte status was determined by microscopic examination with aniline blue staining [20,21]. The *Epichloë*-endophyte-infected plants (E+) were selected for the experiment.

### 2.2. Experimental Design

On 1 August 2015, 20 E+ plants were transplanted into pots (diameter 10 cm, height 15 cm) containing black soil (soil properties: the pH-value was 6.7; the organic carbon content was 121.3 g·kg^−1^; the total nitrogen content was 12.86 g·kg^−1^; the total phosphorus content was 2.1 g·kg^−1^; one seedling per pot). These pots were randomly placed in a greenhouse of Lanzhou University (temperature 25 ± 2 °C; humidity 50% ± 2%; natural daylight). Then, after two weeks of growth, four different water-holding capacities were established, which included severe drought (15% relative saturation moisture content-RSMC), moderate drought (30% RSMC), light drought (45% RSMC) and normal moisture (60% RSMC). Before the test, we weighed the soil in all pots and measured its water content, and each pot was individually watered to a different water content; and then during the experiment, each pot was weighed and watered every evening at 6 p.m. to maintain the appropriate soil moisture content, i.e., at 15%, 30%, 45% or 60% RSMC until the end of the experiment. After three months of growth, the aboveground tissues of all pots were harvested, and all plant samples were freeze-dried (PowerDry LL 3000; Thermo Fisher Scientific, Waltham, MA, USA) and ground into a powder (MM 400; Retsch, Haan, Germany). Then, the concentrations of peramine and lolitrem B in the plant sample were determined.

### 2.3. Determination of Alkaloids

Peramine and lolitrem B concentrations were measured in the aboveground tissue using high-performance liquid chromatography (HPLC) [21]. Peramine and lolitrem B standards were provided by Dr. Wade Mace of Agresearch Ltd., Grasslands Research Centre, Palmerston North, New Zealand.

### 2.4. Data Analysis

Statistical analysis was performed using SPSS 13.0 for Windows (SPSS Inc., Chicago, IL, USA). The effects of drought stress on peramine, lolitrem B, plant height and dry matter (DM) were evaluated using one-way ANOVA. The confidence level was 95%. Values are given as the mean ± standard error (SE).

## 3. Results

**Figure 1:**
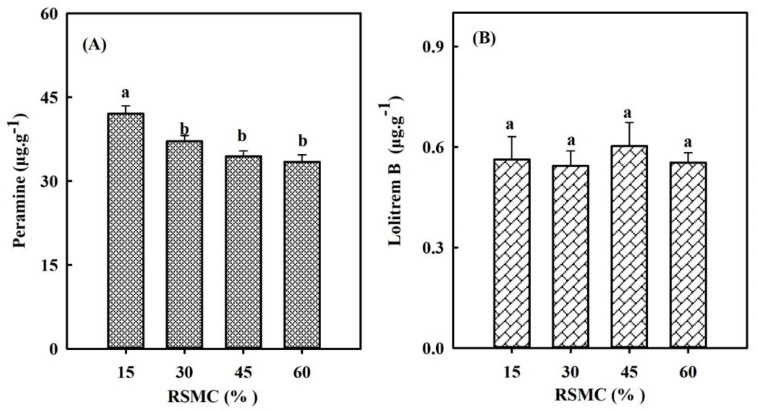
(**A**) Effects of drought stress on peramine concentrations in perennial ryegrass–endophyte symbionts; (**B**) Effects of drought stress on lolitrem B concentrations in perennial ryegrass–endophyte symbionts. Values are given as the mean ± standard error (SE). Different lowercase letters indicate significances differences under different drought stress at *p* < 0.05.

The results showed that the drought stress had a significant effect (*p* < 0.05) on peramine concentration but did not affect the lolitrem B concentration (Figure 1A,B). As drought increased, the content of peramine increased significantly (*p* < 0.05) and peaked at 15% RSMC, but the content of lolitrem B did not change significantly (Figure 1A,B).

**Figure 2:**
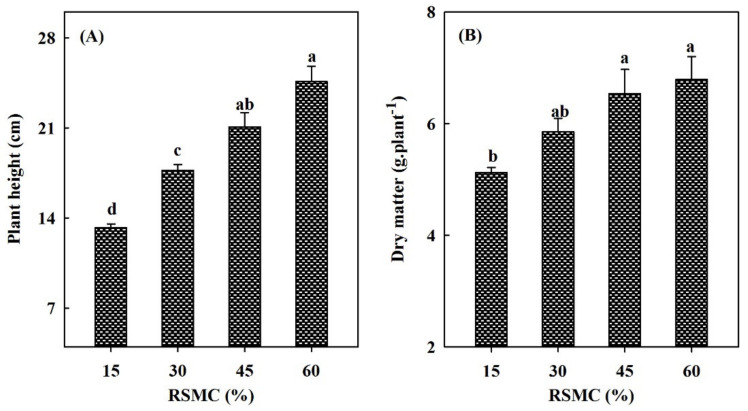
(**A**) Effects of drought stress on plant height of perennial ryegrass–endophyte symbionts; (**B**) Effects of drought stress on dry matter of perennial ryegrass–endophyte symbionts. Values are given as the mean ± standard error (SE). Different lowercase letters indicate significances differences under different drought stress at *p* < 0.05.

The drought stress also significantly (*p* < 0.05) affected the plant height and dry matter of perennial ryegrass (Figure 2A,B). With the increase of drought, the plant height and dry matter weight of perennial ryegrass decreased significantly (*p* < 0.05) and reached the minimum at 15% RSMC (Figure 2A,B).

## 4. Discussion

Having the ability to contribute to pasture sustainability goals, grass–fungal endophyte symbioses are important in pasture systems in China [22,23]. In recent years, the author’s research team has focused on the breeding of perennial ryegrass–endophyte symbionts. It is essential to detect the alkaloids levels of ryegrass under different soil drought conditions.

A previous study showed that water deficit can change the lolitrem B and ergovaline contents of perennial ryegrass–endophyte symbiotic association [17]. Loline alkaloid produced by the *Lolium arundinaceum*–*Epichloë coenophialum* endophyte symbiont was increased with increasing water deficit [24]. Ergonovine and ergine alkaloids produced by *Achnatherum inebrians*–*Epichloë gansuense* symbionts were increased with increasing drought and salt stress [25]. However, the results of research on the relationship between alkaloid content and soil moisture are currently inconsistent. Another study showed that the content of peramine alkaloid did not vary in *Epichloë*-endophyte-infected *Festuca arizonica* (Vasey) under different soil moisture conditions [26]. Our results showed that the peramine concentration produced by endophyte-infected perennial ryegrass peaked under severe drought (15% RSMC), while the lolitrem B content did not differ with different soil drought treatments. These results are similar to those of previous studies. Peramine is a good natural insecticide [27], when the soil appears dry, the increase of peramine in endophyte-infected perennial ryegrass could help plants against herbivores. However, the lolitrem B alkaloid content was high, and its presence in endophyte-infected ryegrass may cause livestock poisoning [21,28]. In addition, our results also showed that the plant height and dry matter of endophyte-infected perennial ryegrass decreased with the increase of drought, which was consistent with previous research results.

We concluded that drought stress increased the concentration of peramine produced by the symbiont but did not affect the content of lolitrem B.

## Data Availability

Not applicable.
